# The pyeTribe: Simultaneous eyetracking for economic games

**DOI:** 10.3758/s13428-016-0819-9

**Published:** 2016-10-28

**Authors:** Tomás Lejarraga, Michael Schulte-Mecklenbeck, Daniel Smedema

**Affiliations:** 1 Center for Adaptive Rationality, Max Planck Institute for Human Development, Lentzeallee 94, 14195 Berlin, Germany; 2 Department of Business Administration, University of Bern, Bern, Switzerland; 3 Indiana University, Bloomington, Indiana USA

**Keywords:** Eyetracking, Simultaneous, Public goods game, Attention

## Abstract

The recent introduction of inexpensive eyetrackers has opened up a wealth of opportunities for researchers to study attention in interactive tasks. No software package has previously been available to help researchers exploit those opportunities. We created “the pyeTribe,” a software package that offers, among others, the following features: first, a communication platform between many eyetrackers to allow for simultaneous recording of multiple participants; second, the simultaneous calibration of multiple eyetrackers without the experimenter’s supervision; third, data collection restricted to periods of interest, thus reducing the volume of data and easing analysis. We used a standard economic game (the public goods game) to examine the data quality and demonstrate the potential of our software package. Moreover, we conducted a modeling analysis, which illustrates how combining process and behavioral data can improve models of human decision-making behavior in social situations. Our software is open source.

Economic games are the experimental instrument primarily used by economists and psychologists to study how individuals make decisions while interacting with others. Careful design of economic games allows researchers to infer people’s motives and decision processes from observing their choices. Recently, however, researchers have moved from studying choices alone to a more process-focused perspective (Camerer & Johnson, 2004; Fehr & Schmidt, 1999; Schulte-Mecklenbeck, Kühberger, & Ranyard, 2011; Wang, Spezio, & Camerer, 2010). For example, they have begun using various tools to track attention. One example is the use of MouseLab to monitor people’s information acquisition when making decisions (Willemsen & Johnson, 2011). Although MouseLab has given researchers a window into the decision-making process, some have argued that proceeding through information within this framework, in which information is hidden behind boxes, incurs costs that might influence decision processes and, ultimately, choices (Glöckner & Herbold, 2011; Lohse & Johnson, 1996). Eyetrackers come without the problem of extensive search costs, as information is available literally at a glance.

There is, however, a downside to eyetracking, one that is particularly important when studying interactive games: Given the hefty initial price tag of tens of thousands of US dollars per unit, few research institutions have previously been able to afford more than one eyetracker, if any at all. Relying on a single eyetracker is particularly problematic in the context of interactive economic games, in which researchers are interested in an online account of how participants react to the behavior of others. A single eyetracker allows researchers to track the attention of one player, but the simultaneous dynamics of attention during group interactions remains hidden.

The recent introduction of inexpensive eyetrackers is changing this landscape considerably. One example is an eyetracker marketed by The Eye Tribe (www.theeyetribe.com) for $99, or a Tobii (www.tobii.com) for $200. Other companies, including SMI (www.smivision.com), are also beginning to offer trackers in a much lower prize range (typically around $500, as of 2016). With this substantial reduction in prices, laboratories are now able to acquire *several* units, enabling the simultaneous eyetracking of multiple participants. To date, however, experiments involving multiparticipant interaction and multiple eyetrackers have not intersected. To our knowledge, no available software package allows researchers to construct an experiment to simultaneously collect behavioral and eyetracking data from (many) interacting participants. We created such a software package.

## Synchronizing by matching time stamps

A simple, yet tedious approach to conducting simultaneous eyetracking studies is to run a behavioral experiment using existing software (e.g., zTree) while recording eyetracking data individually on multiple computers at the same time (see, e.g., Fiedler, Glöckner, Nicklisch, & Dickert, 2013). Matching the time stamps from eyetracking and behavioral data, without accessing the eyetracker controls, may provide the researcher with the desired datasets for analysis. However, we find this approach inadequate for three reasons.

1. Gaze-contingent experiments, in which the procedure is conditioned on specific gaze patterns, are not possible without access to the tracker data in real time. In a multiparticipant setup, the experimenter may want to make one participant’s display contingent on the participant’s own gaze location, on another participant’s gaze location, or some combination of the two. For example, the experimenter may want participants to proceed to make decisions only when all interacting participants have fixated on all relevant information. This cannot be accomplished by matching time stamps, but only by processing tracker data in real time.
2. Without direct access to the eyetracker controls, calibration and other tasks must be conducted independently of the behavioral task and be supervised by the experimenter. In experiments with many participants, this is costly, and participant–experimenter interaction introduces noise. With direct access to the tracker controls, the experimenter can decide a priori how to perform the calibration, and can run it in a self-paced manner. For example, the experimenter can decide under which conditions the calibration will be considered successful, the number of calibration trials, and the procedure to follow if calibration fails (e.g., to continue the experiment with or without tracking, to quit the experiment, or to replace the participant by one whose calibration was successful). In such a setup, the eyetracker can be calibrated without the experimenter’s supervision and simultaneously for several participants.
3. Matching time stamps generates an unnecessarily large amount of data, because the recording of information cannot be turned on and off at desired/synchronized points of the experiment. Using a centralized software package to bundle the communication of each participating client allows control of the eyetracker in terms of onset and offset in recording periods of interest. Onset and offset signals may also be contingent on specific behaviors in the experimental task, or on specific fixation patterns. This allows the more selective and efficient collection of data.

## Features required in the software package

In developing software to coordinate experiments with multiple participants in eyetracking studies, we first identified the following features that would be necessary in the package:

1. *User input*: Record mouse clicks, keystrokes, or other input that participants use to express preferences or valuations.
2. *Eyetracker data*: Record time-series data generated by the eyetracker, including *x*,*y* coordinates of gaze locations on the screen and pupil dilation.
3. *Network data*: Coordinate user input, eyetracker data, and other information transmitted by instances of the software running on all participants’ computers.
4. *Scalability*: Scale easily to *n* participants (where *n* > 2).

Items 1–3 relate to data-handling issues. Some existing software packages handle these three types of data independently. For example, experimental software packages commonly used in psychology or neuroscience (E-Prime, www.pstnet.com/eprime.cfm; Open Sesame, www.osdoc.cogsci.nl) handle mouse and keyboard inputs with the appropriate drivers; several other applications are able to process eyetracker data (E-Prime; Presentation, www.neurobs.com; Psychophysics Toolbox in MATLAB, www.psychtoolbox.org) in various setups, and even network data (zTree; Fischbacher, 2007) in economic games. Although the above-mentioned software packages can handle some of the listed features, currently no software package is available that can process the flow of all these data sources *natively* and *in real time*.

Item 4, scalability, becomes important when it is necessary to adapt to changing experimental situations (e.g., different numbers of participants per experimental session). One way to achieve a scalable setup is to centralize certain processes on a server and to let clients communicate with this central node in a so-called star network (Roberts & Wessler, 1970). In a star network with *n* connections, each client connects to one network location, rather than to the addresses of every other client. Hence, the software on the server has the flexibility to deal with sessions with different numbers of participants. For example, if the laboratory has 20 computers prepared for the experiment but only 18 participants show up, the experiment can still be conducted, because the server will automatically account for the incoming network connections.

An additional advantage to this design is that the software running on the server can take a more active role than simply relaying information. If an experiment requires a calculation to be made using input from multiple users (e.g., the sum of participants’ contributions to a common pool), the server can perform such calculation and immediately send the result back to all clients.

## Application example: Iterated public goods game

One paradigmatic example of economic games is the public goods game (PGG). Although various theories of social behavior accurately predict the aggregate data obtained in a PGG, their psychological plausibility has been questioned. Recent research attempted to uncover the mechanisms underlying decisions in social dilemmas—that is, the way information is processed to make decisions in the social context (Fiedler et al., 2013). In what follows, we briefly describe an experiment in which we used the Eye Tribe eyetracker in combination with our software package to run an iterated PGG. We had two goals. First, we sought to evaluate the quality and integrity of data stored by our software package. Second, we examined the capability of our software package to handle interactive decision-making experiments.

## Method^1^

### Apparatus

**Fig. 1:**
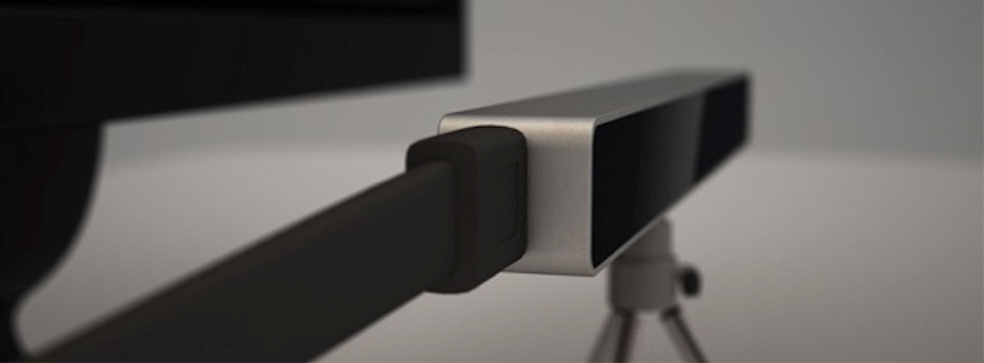
Eye Tribe tracker with USB plug in front of a computer monitor

Eye movements were recorded using an Eye Tribe tracker (see Fig. 1), which records binocular gaze data with a sampling rate of 60 Hz and an accuracy of 0.5°. The software provides dispersion-based fixation data for further analysis.

**Fig. 2:**
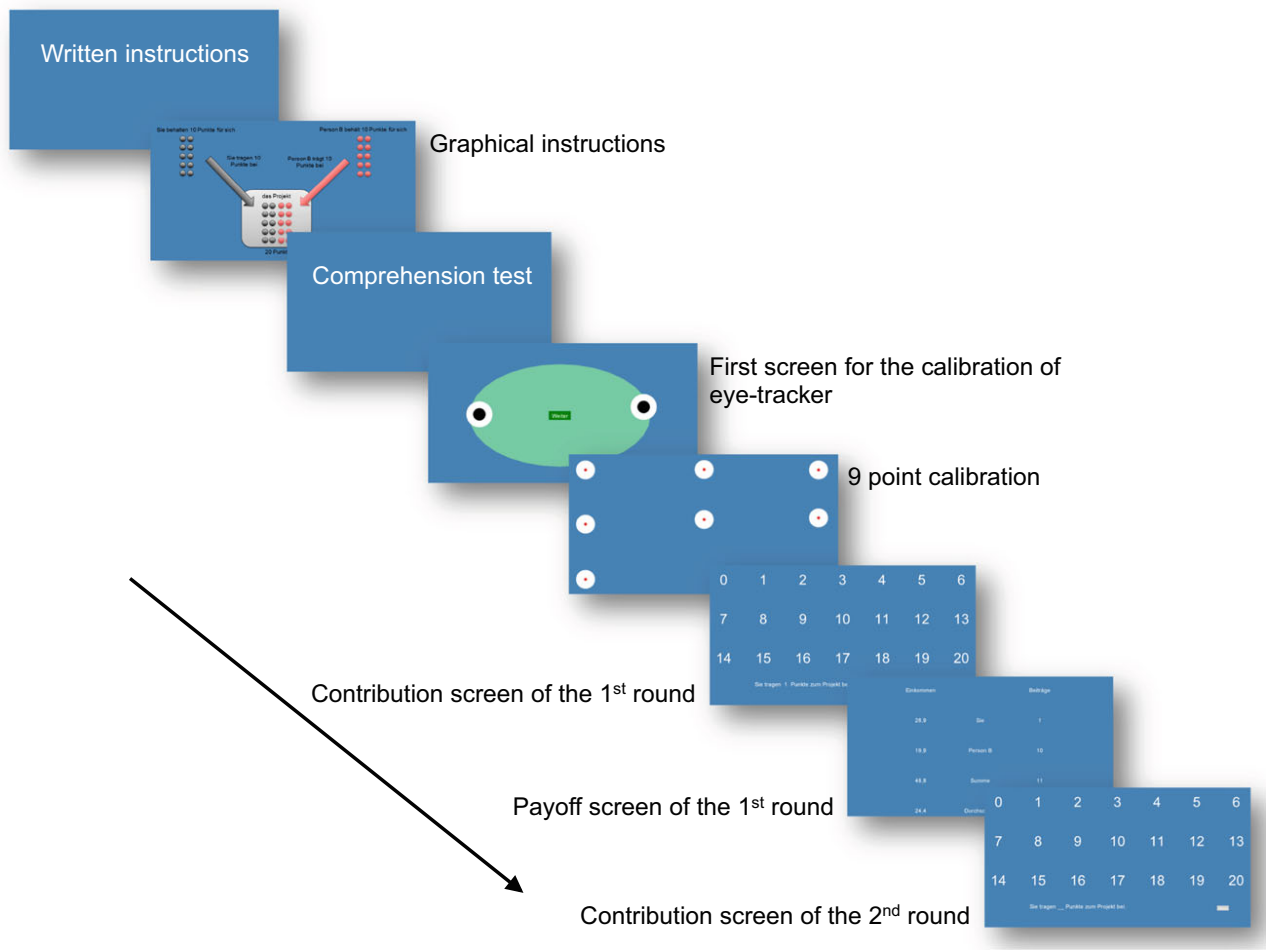
Sequence of experimental screens presented to the participants. After a set of written/graphical instructions and a comprehension test, the eyetracker is calibrated with a 9-point calibration. After successful calibration, participants see the first-round contribution screen with all possible contributions [0–20]. The following response screen shows the participant’s and the other player’s (Person B) contribution, as well as summary statistics (sum, average) for the current game. The maximum possible, nonoverlapping square areas around the numbers (contribution screen) and alphanumeric characters (response screen) were used as the AOIs

The participants were seated in front of screens with a resolution of 1,920 × 1,080 pixels on computers with Windows 7. Nonoverlapping areas of interest (AOIs) around the numeric information on screen were defined, with a size of 400 × 200 pixels (see Fig. 2).

Each participant was calibrated using the 9-point calibration procedure provided in the Eye Tribe development kit. To this end, participants were asked to look at points appearing sequentially in different locations on a dark computer screen. If sufficient quality was reached (i.e., accuracy of <0.5°), the experiment was started. If the visual angle was >0.5°, recalibration was triggered automatically.

## Results

### Data quality

Every participant in our experiment successfully completed the calibration procedure in less than three calibration attempts, our cutoff criterion for exclusion from the study. To evaluate the quality of the data collected, we ran two simple tests: (a) correspondence between the last number fixated by a participant on the contribution screen and that participant’s actual contribution; and (b) AOI versus non-AOI fixations on the feedback screen.

**Fig. 3:**
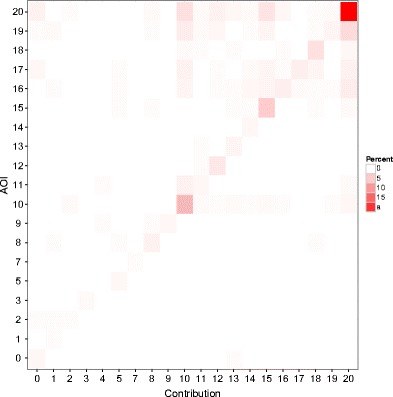
Correspondence between the last fixation of an AOI and the contribution chosen via mouse click in this round

Participants were asked to pick a number from 0 to 20 on the contribution screen using their mouse. These numbers were presented in matrix format across the whole screen (see Fig. 2, sixth screen). Consistent with previous results showing that mouse and eye movements are highly correlated (Chen, Anderson, & Sohn, 2001), we found high correspondence^2^ between the last fixation on an AOI and the number that the participant selected and clicked on (see Fig. 3) for the large contributions (10–20). However, this pattern was weaker for small contributions (0–9). The difference in the correspondences between high and low contributions is partly explained by the fact that contributions tended to be relatively high in general: The median contribution in Round 1 was 12, with only four cases (out of 120) below 10 (see the analysis below of the contribution patterns for more details). For the quadrant with the highest contributions in Fig. 3 (20, 20), we observed that the last fixation in an AOI showed a correspondence with the actual choice (i.e., click) on the same AOI for 24.7 % of all clicks in Game 1.

A second indication of the data quality is the relation of the fixations within AOIs versus non-AOIs (but see Orquin, Ashby, & Clarke, 2016, on flexibility in the definition of AOIs and the consequences for data analysis). Our feedback screen consisted of eight AOIs with numeric information and six AOIs with written text (see Fig. 2, Screen 7). Across all participants, 28 % of fixations were in numeric areas, and 69 % were in the written text. About 3 % of all fixations were outside a defined AOI,^3^ most likely related to participants’ orientation on the stimulus screen or reflecting incorrect classifications of a fixation to a non-AOI area.

### Process analysis

**Fig. 4:**
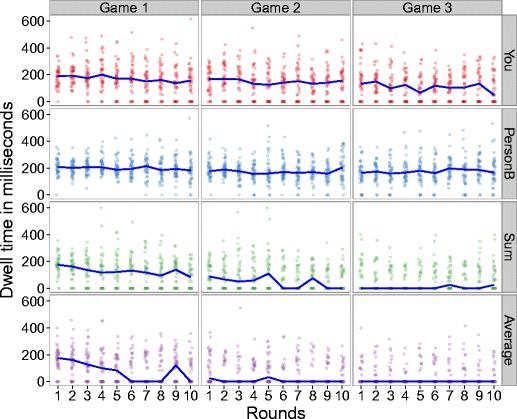
Median dwell times for each game (1–3), round (1–10), and AOI (You, PersonB Sum, and Average)

On average, participants fixated on 17 AOIs (*SD* = 9 AOIs), which took them 92.9s (*SD* = 42.3 s). To evaluate the attention to different aspects of the PGGs played, we calculated the dwell time (sum of all fixation durations during a dwell—i.e., one visit to an AOI from entry to exit, measured in milliseconds; Holmqvist et al., 2011, p. 190) separately for each participant, each game (1–3), and each round (see Fig. 4).

The players’ attention was focused primarily on two AOIs: their own points (the two AOIs in the upper row of Fig. 2, indicated by “You”) and the points of the other player (the two AOIs in the second row of Fig. 2, indicated by “Person B”). Whereas the attention given to “You” (a player’s own points) decreased across games, to 0 in Game 3, the attention dedicated to “Person B” remained relatively constant across games. In contrast, the information available on both the sum score and the average attracted less attention from players; the median dwell time converged to 0 early on, and remained unchanged across Games 2 and 3. We qualified these descriptive results with a mixed-effect model approach, using the lmer function of the lme4 package (Bates, Maechler, Bolker, & Walker, 2014) in R. Significance tests were conducted using the lmertest function of the lmerTest package. Participants were modeled as random intercepts; AOIs and rounds were added as random slopes to account for the repeated measures nature of the data (Barr, Levy, Scheepers, & Tily, 2013). We found significant main effects of the time spent on an AOI, *F*(5, 12510) = 5.86, *p* = .001, with the following average dwell times on AOIs: *M*_PersonB_ = 192.6 ms (*SD*_PersonB_ = 120.9 ms), *M*_You_ = 196.7 ms (*SD*_You_ = 128.5 ms), *M*_Sum_ = 175.2 ms (*SD*_Sum_ = 114.4 ms), *M*_Avg_ = 181.1 ms (*SD*_Avg_ = 115.2 ms). Longer dwell times were found for information about the other player (Person B) and the player’s own information. Summary statistics (mean and sum) received shorter dwell times. Furthermore, round, *F*(1, 6959) = 10.1, *p* = .001, and game, *F*(1, 4.5) = 9.9, *p* = .03, both showed main effects [*M*_Game1_ = 199.2 ms (*SD*_Game1_ = 125.7), *M*_Game2_ = 181.3 ms (*SD*_Game2_ = 118.1 ms) *M*_Game3_ = 179.4 ms (*SD*_Game3_ = 115.2)], indicating faster dwell times at the end than at the beginning of the experiment. None of the interactions reached significance.

### Models of contributions

We modeled individual contributions (see the analysis of the contribution patterns in Appendix B) trial by trial using conditional cooperation strategies (for details, see Fischbacher & Gächter, 2010). Specifically, we used a perfect conditional cooperator with naïve beliefs and a perfect conditional cooperator with actual beliefs—the other strategies proposed by Fischbacher and Gächter do not apply in our experimental design. We also proposed a simple matching model. Our matching model assumes that the contribution *C* of an individual *i* at trial *t* is a weighted average of the previous contribution of *i* (*C*_*i*,*t–1*_) and the previous contribution of the other player *j* (*C*_*j*,*t–1*_). Formally, the contribution in each trial is

1 $$ {C}_{i,t}={w}_i\left({C}_{i,t-1}\right)+\left(1-{w}_i\right)\left({C}_{j,t-1}\right), $$

where *w*_*i*_ is the weight given to the player’s own previous contribution, and 1 *– w*_*i*_ is the weight given to the other player’s previous contribution. The parameter *w* can be interpreted as capturing the relative attention devoted to a player’s own versus the other player’s payoffs.

We first considered a matching model with *w* set at .5—that is, a model that simply averaged the previous contributions of the two players. We then compared the performance of the matching model with that of the two conditional cooperation strategies by calculating the mean squared deviation (MSD) between each model and individual behavior. We found that our matching model predicted individual behavior more accurately than did the two conditional cooperation strategies, with MSDs of 20.7, 17.3, and 15.8 for perfect conditional cooperation with naïve beliefs, perfect conditional cooperation with perfect beliefs, and the matching model, respectively. We then estimated an individual *w* parameter for each participant, attempting to minimize the MSD between the predictions of the model and individual behavior. The result of the fitting procedure was a *w*_*i*_ corresponding to each individual. If *w* captures attention, we should expect to observe a correlation between the fitted *w*s and the relative attention given by players to each other’s information, as measured by eyetracking.

We therefore calculated a measure of relative attention as a ratio of the sums of fixations distributed between player *i*’s own information and player *j*’s information, as displayed in the payoff screens for each round. The relative attention *ra* across the experiment for player *i* was

2 $$ r{a}_i=\frac{{\displaystyle \sum fi{x}_i}}{{\displaystyle \sum fi{x}_i+{\displaystyle \sum fi{x}_j}}} $$

**Fig. 5:**
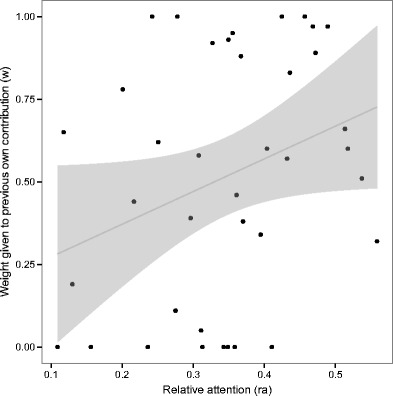
Positive relations between relative attention (ra) and the weight given to the player’s own previous contribution (*w*), derived from fitting the matching model to each individual

The correlation between *w* and *ra* across individuals was .31, *p* = .055. As Fig. 5 shows, *w* and *ra* were positively related.

**Fig. 6:**
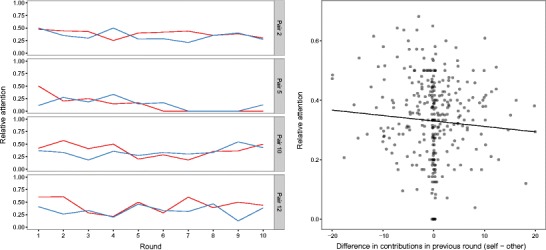
(Left) Evolution of relative attention (Eq. 1) within each of four selected pairs of players. (Right) Relative attention as a function of the difference between a player’s own contribution and that of the other player. The negatively sloped regression line indicates that the more a participant contributes relative to the other participant, the more the participant pays attention to the information concerning the other player

The data collected with pyeTribe allowed us to examine whether and how the attention between players in a PGG co-evolves. The left panel of Fig. 6 shows the evolution of relative attention (*ra*, as defined in Eq. 1) for four selected pairs of players. The patterns of relative attention are diverse. For the top two pairs in the panel, attention seems to converge across rounds. For the bottom two pairs, in contrast, attention seems more erratic. Naturally, players do not observe each other’s attention, but only their contributions. Therefore, a more reasoned examination of how attention evolves would require an analysis of the contributions, which are the only information transmitted between players. The right panel of Fig. 6 attempts to shed some light on how relative attention responds to differences in the contributions. As the right panel shows, the larger the difference between a participant’s own contribution and that of the other player, the more attention a player pays to the other participant.

## Discussion

New research tools are opening up a wealth of opportunities for researchers to study new paradigms and ask new questions. Eyetrackers are, of course, not new, but the critical reduction in price is allowing for new experimental setups to study, among other things, attention in interactive decision-making tasks. In this article, we presented a software package designed to help researchers exploit the advantages of multiple eyetrackers while conducting interactive decision-making experiments.

Our software package has four central features. First, a large number of participants can be recorded while they interactively play economic games. Second, eyetracking calibration can be performed without the experimenter’s supervision, allowing for simultaneous calibration, saving significant resources. Third, gaze-contingent rules—for instance, proceeding to the next screen only when all interacting participants have fixated on a particular AOI—allow for complex experimental scenarios to be designed. Fourth, there is a clear potential to integrate our software package into more complex experimental systems, like BoXS (Seithe, Morina, & Glöckner, 2015) or zTree (Fischbacher, 2007).

We used a standard economic game (PGG) to demonstrate the potential of our software package and to examine the quality of the data obtained. The value-for-money ratio of the eyetracking data collected was outstanding, as shown by the high correspondence between fixations at the moment of clicking the mouse and the number selected, and the high proportion of fixations within AOIs. A modeling analysis of the individual contribution processes provided further evidence for the high quality of the data. We proposed a simple model that weight-averages a player’s own previous contribution with that of the other player. Specifically, we estimated the weight that each participant assigned to his or her own previous contributions and examined whether those weights corresponded with the attention patterns. The relative numbers of fixations between self and other were moderately correlated with the parameters estimated individually. This modeling analysis illustrates how combining behavioral and process data may improve cognitive models of behavior. Fiedler et al. (2013) demonstrated the value of adding process data to the analysis of economic games. We extended their work by facilitating the synchronous observation of multiple players in a PGG (our approach can, of course, be extended to other economic games or experimental conditions involving interactions between multiple players). Importantly, our approach makes it possible to record and examine information search before a choice is made.

Our software package (as well as the data and analysis code) is open source and can be pulled from the following git repository: https://github.com/michaelschulte/ThePyeTribe.

## Figures and Tables

**Fig. 7:**
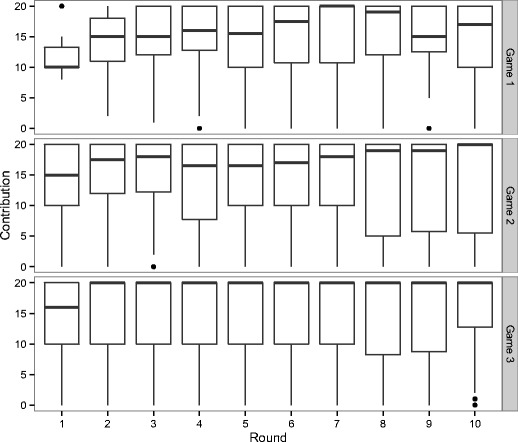
Distribution of contributions for each game (1–3) and round (1–10). The thick black line in each box indicates the median of the distribution; the ends of the boxes indicate the 25 % and 75 % percentiles of the distribution; the whiskers indicate the lowest datum within 1.5 interquartile ranges (IQR) of the lower quartile and the highest datum within 1.5 IQR of the upper quartile. Points indicate outliers—that is, data beyond 1.5 IQR

## Appendix A

### Application example: Method

#### Participants

Forty students (21 female, 19 male) participated in our experiment in groups of two. The study was run at the Max Planck Institute for Human Development, Berlin. The mean age of participants was 25.1 years (*SD* = 3.7 years), and they received a fixed hourly fee of €10 and a bonus depending on their choices in a randomly selected round of the game. Participants provided written consent according to the institute’s ethics guidelines. The experiment took about 60 min. All participants had normal or corrected-to-normal eyesight and were instructed to find a comfortable sitting position with a straight view to the monitor/Eye Tribe camera.

#### Material and procedure

We used a standard iterated PGG (Camerer, 2003). The participants were anonymously and randomly assigned into pairs. The members of each pair were seated in different rooms. Participants were provided with the instructions for a two-person PGG (Reuben & Riedl, 2009), and their understanding of the game was tested by comprehension questions. They were informed that all participants had the same instructions.

Each participant was endowed with 20 points and had to decide how many to contribute to a public good. Participants decided anonymously and simultaneously. The sum of participants’ contributions was multiplied by a factor of 1.8 (i.e., an interest rate of 80 %), and the resulting amount was shared equally between the two players. Each participant’s payoff was then determined—namely, the amount kept (not contributed to the public good) plus the amount received from the public good:

$$ \mathrm{Payoff}=20-\mathrm{own}\ \mathrm{contribution}+1.8 \times \frac{\mathrm{sum}\ \mathrm{of}\ \mathrm{contribution}\mathrm{s}\ }{2}. $$

After deciding on their contributions, participants saw a feedback screen showing information on both their and the other player’s contributions and earnings. Each participant played three games with the same partner. Each game involved ten rounds (i.e., ten contribution decisions). We used different multiplication factors in the three games. In the first game, the multiplication factor was set at 1.8; in the other two games, the factors were 1.5 and 1.3, in random order.

The experimental sessions were coordinated by means of a centralized “handler” that paced the PGG and the eyetrackers. This handler coordinated and checked the calibration of the eyetrackers (on the basis of data provided by the Eye Tribe application programming interface), and ran the PGG.

### Data cleaning

The eyetracking data were cleaned in two steps: (1) at the lower bound of the dataset, very short acquisitions (<50 ms) were removed, because they most likely represented spurious eye movements with no information acquisition, and (2) at the upper bound of the dataset, very long acquisitions (two *SD*s above the average fixation length) were also removed.

### Contribution analysis

Behavior in PGGs is usually tested against the Nash equilibrium. In games in which the multiplier is between 1 and the number of interacting players, the Nash equilibrium equals “no contributions” to the public good. However, such a pattern of behavior is rarely observed in single-play PGGs (Zelmer, 2003). In iterated PGGs like the one used in our experiment, participants approach equilibrium (Levitt & List, 2007). A number of factors have been found to increase contributions away from equilibrium. For example, in “open” PGGs, in which participants receive feedback about the amount contributed by each player (as in the present experiment), contributions are higher than when personalized feedback is not provided. The magnitude of the multiplication factor also has a significant impact on the levels of contributions, with higher factors producing higher contributions (Zelmer, 2003).

We examined participants’ contributions across rounds in each of the three games. The conditions we used in our experiment (i.e., “open” feedback and high multiplication factors) were expected to produce high contributions. Figure 7 shows the patterns of these contributions. As predicted, we found high levels of contribution that increased across rounds and games.

## Appendix B

### The Eye Tribe application program interface (API)

In what follows, we describe how our software package is connected to the Eye Tribe API. A more complete description of the API can be found at http://dev.theeyetribe.com/api/.

The hardware requires a USB 3.0 connection to the host computer, as well as installation of the Eye Tribe Server software. A transmission control protocol socket is opened in order to send and receive data from the tracker, making it possible to retrieve eyetracking information and send commands during the experiment itself. Eye Tribe Server requires any software package connected in this way to send frequent keep-alives, called “heartbeats,” every 250 ms. If a heartbeat is not received for 2 s, the Eye Tribe Server will terminate the connection.

The Eye Tribe API allows a variety of data to be sent and received during runtime. For example, the sampling frequency can be set to 30 or 60 Hz through command-line parameters to Eye Tribe Server or through a configuration file, but this setting cannot be changed without terminating the Eye Tribe Server process and restarting the software package.

The API specifies the use of JavaScript Object Notation (JSON) strings to communicate with the tracker. Every message the user sends receives a reply. The Eye Tribe Server can send data without a request under two circumstances: (a) if certain parameters change (e.g., the calibration state), an automatic message will be sent; (b) the user can set the tracker to “push” mode, which means that it will automatically send new eyetracking data as soon as they are available. These snapshots are referred to as “frames,” and contain information including pupil size and the location for each eye, the gaze location for each eye, an average across both eyes, gaze locations smoothed using the data from several frames across time, and timestamps.

#### The pyeTribe

The pyeTribe package provides a Python interface for the hardware. It is not intended to replace working knowledge of the Eye Tribe API, but rather to supplement it. PyeTribe has the following features:

- ***HeartThread*** A thread that continuously sends heartbeats—that is, keep-alives—to the Eye Tribe Server.
- ***ListenerThread*** A thread that continuously listens for messages from the Eye Tribe Server. It splits the data stream into individual messages and places these in a queue to be processed.
- ***ProcessorThread*** A thread that sorts the data received by the ListenerThread and then takes appropriate actions. If the message is an update notification, any piece of code that is waiting for that update will be notified. If the message is frame data, it will be written to a file, should one be specified; otherwise, only the most recent frame will be saved. If the message is an acknowledgement that a heartbeat was received, it will simply be discarded. If the message is a reply to a user-sent message (e.g., a get request or a request to start a calibration), it will be placed in a data structure called an EyeTribeQueue.
- ***EyeTribeQueue*** This is a queue that inherits from the LifoQueue from Python’s built-in Queue module. We used this approach because we aimed to obtain a thread-safe, searchable data structure, and LifoQueue comes with the machinery for thread safety as well as for storing data in a basic list that is easily searchable. We overrode LifoQueue’s get method with one that will only produce an error, and created a new get_item method that takes certain parameters about the original request as arguments and finds the correct reply to the given request.
  The ProcessorThread needs to sort the incoming messages, because Eye Tribe Server’s behavior is not completely predictable from pyeTribe’s point of view. The code is designed so that the connection is accessed only one thread at a time; the Eye Tribe Server may send a different type of data. For example, if a request is sent to get the current frame rate, and the tracker is unexpectedly unplugged at the same time, the next message received might be an update that the tracker state has changed. We used EyeTribeQueues so that when a request is sent, the pyeTribe waits for an appropriate reply to be received, and then removes that reply from the queue and returns it to the function that sent the request.
- ***Main class*** 4 When initialized, this class automatically opens a connection to the Eye Tribe Server using the default host and port; it starts the HeartThread, ProcessorThread, and ListenerThread, and creates the queues they will be using to share data.

The following code creates an instance of the main class called “et,” whose methods can be called to control the tracker’s behavior and obtain information.

      from thepyetribe import EyeTribeServer

      et = EyeTribeServe *r* ()

Python “property” decorators are a convenient way to interact with the tracker’s variables. Properties are similar to private data members with accessor methods. The user specifies what code is to be run when the property is accessed, and what code is to be run when the property is assigned a value. This means that instead of writing commands linked with “and”—for example, et.push.set(True) and if et.push.get()—the code can be simplified: et.push = True or if et.push.

Every variable specified by the Eye Tribe API gets a property in the main class. Those that are immutable will raise an exception if the user attempts to assign different values to them.

We provide methods for calibration, though the user must manually choose how many calibration points to use and the coordinates of each.

We also provide a method to estimate the time difference between the internal clock of the computer and that of the tracker. This method measures a combination of the actual differences between the two clocks and tracker latency. The latency should be less than 30 ms, but is usually less than 16 ms. If latencies need to be within one frame, this function provides an important check.

Finally, the main class has a method called record_data_to, which allows the user to change where data are recorded at any time. Calling record_data_to(None) will stop all recording, but the most recent frame will still be saved in the software’s memory and remain constantly updated and accessible.

## Appendix C

### Setting up your computer to run pyeTribe

1. Install the Eye Tribe Server.
  You will need to create an account with The Eye Tribe to download the Eye Tribe Server software; follow their instructions for the installation.
  https://theeyetribe.com/
2. Install Python 2.
  Install any version higher than 2.7.6 and lower than 3.0. This guide assumes you will use the default installation settings.
  https://www.python.org/downloads/
3. Install PyYAML.
  At a command prompt, enter “pip install pyyaml” after installing Python.
  http://pyyaml.org/wiki/PyYAML
4. Install PsychoPy, along with all its dependencies.
  Probably the easiest way to do this is to download the “Standalone” PsychoPy version and merge PsychoPy2/Lib/site-packages into Python27/Lib/site-packages (i.e., after the install, copy the contents of PsychoPy2/Lib/site-packages and paste them into Python27/Lib/site-packages; if there are any conflicts, keep the original files in Python27/Lib/site-packages).
  https://github.com/psychopy/psychopy/releases
5. Install Git.
  https://git-scm.com/downloads
6. Install the experiment.
  Create a folder that you want the experiment to go in. Open the Git Bash to that folder, and enter the following command (all one line):
  An easy way to set up multiple computers is to copy and paste the Python files installed on the first computer to the other computers (you do not have to repeat Steps 5–8 separately for each computer).
7. Copy the handler.py file to the computer on which you want to run it. Any lab computer or server in your local area network that has Python installed can run the handler. Note the IP address of the handler’s computer, and make sure that incoming traffic is allowed on port 11111. You only need to do this step once.
8. Open the EyeGames.cfg file in a text editor.
  Specify the numbers of players, rounds, and games, and the handler IP. In the following example you will find the header of the config file for one two-player game with ten rounds. A PGG multiplier of 1.8 is used for this round (see the main text), and the location of the handler is set to the local machine (localhost). Note that you would have to specify the IP address of the handler on the other machines here!

### Running the experiment

- Start the handler (open a command prompt to the handler’s folder and enter “python handler.py”).
- On each computer, plug in the Eye Tribe tracker and start the Eye Tribe Server. You can use the icon generated during the setup to open the Eye Tribe in 30-frame-per-second mode, or you can open it from the command prompt and specify the frame rate there. For a 60-frame-per-second setup, you would enter:
  eyetribe --framerate=60
- On each computer, begin the experiment by either double-clicking on main.py or entering into a command prompt (within the experiment folder): python main.py
- Select “experiment” mode for the full experience, or “demo” mode for an experience run in a window. “Debug” mode allows developers to quickly reach any given point in the experiment’s flow.

## Footnotes

1 You will find information about the participants, materials, and procedure in Appendix A.

2 We operationalized “correspondence” as the percentage of matches between the last fixation and the selection of a contribution. A low percentage means that after the last fixation of an AOI, the actually chosen contribution was different in most cases; a high percentage means that in many cases the last fixation on an AOI was followed by actual choice of the corresponding contribution. The analysis presented here was only done with the data from Game 1, but similar patterns emerged for all three games.

3 Note that 56 % of the area of the screen was covered by AOIs.
